# Initial and Residual 3D Fracture Displacement Is Predictive for Patient-Reported Functional Outcome at Mid-Term Follow-Up in Surgically Treated Tibial Plateau Fractures

**DOI:** 10.3390/jcm12186055

**Published:** 2023-09-19

**Authors:** Nick Assink, Eelke Bosma, Anne M. L. Meesters, Sven H. van Helden, Robert J. Nijveldt, Kaj ten Duis, Max J. H. Witjes, Jean-Paul P. M. de Vries, Joep Kraeima, Frank F. A. IJpma

**Affiliations:** 1 Department of Trauma Surgery, University Medical Center Groningen, University of Groningen, 9713 GZ Groningen, The Netherlands; a.m.l.meesters@umcg.nl (A.M.L.M.); k.ten.duis@umcg.nl (K.t.D.); 2 3D Lab, University Medical Center Groningen, University of Groningen, 9713 GZ Groningen, The Netherlands; m.j.h.witjes@umcg.nl (M.J.H.W.); j.kraeima@umcg.nl (J.K.); 3 Department of Trauma Surgery, Martini Hospital, 9712 CP Groningen, The Netherlands; eelke.bosma@mzh.nl; 4 Department of Trauma Surgery, Isala Hospital, 8025 AB Zwolle, The Netherlands; s.h.van.helden@isala.nl (S.H.v.H.); r.j.nijveldt@isala.nl (R.J.N.); 5 Department of Surgery, University Medical Center Groningen, 9713 GZ Groningen, The Netherlands; j.p.p.m.de.vries@umcg.nl

**Keywords:** tibial plateau fracture, three-dimensional, 3D, Q3DCT, patient-reported outcome, KOOS

## Abstract

Background: Conventional measures of fracture displacement have low interobserver reliability. This study introduced a novel 3D method to measure tibial plateau fracture displacement and its impact on functional outcome. Methods: A multicentre study was conducted on patients who had tibial plateau fracture surgery between 2003 and 2018. Eligible patients had a preoperative CT scan (slice thickness ≤ 1 mm) and received a Knee Injury and Osteoarthritis Outcome Score (KOOS) questionnaire. A total of 362 patients responded (57%), and assessment of initial and residual fracture displacement was performed via measurement using the 3D gap area (mm^2^). Patients were divided into four groups based on the 3D gap area size. Differences in functional outcome between these groups were assessed using analysis of variance (ANOVA). Multiple linear regression was used to determine the association between fracture displacement and patient-reported outcome. Results: Functional outcome appeared significantly worse when initial or residual fracture displacement increased. Multivariate linear regression showed that initial 3D gap area (per 100 mm^2^) was significantly negatively associated with all KOOS subscales: symptoms (−0.9, *p* < 0.001), pain (−0.0, *p* < 0.001), ADL (−0.8, *p* = 0.002), sport (−1.4, *p* < 0.001), and QoL (−1.1, *p* < 0.001). In addition, residual gap area was significantly negatively associated with the subscales symptoms (−2.2, *p* = 0.011), ADL (−2.2, *p* = 0.014), sport (−2.6, *p* = 0.033), and QoL (−2.4, *p* = 0.023). Conclusion: A novel 3D measurement method was applied to quantify initial and residual displacement. This is the first study which can reliably classify the degree of displacement and indicates that increasing displacement results in poorer patient-reported functional outcomes.

## 1. Introduction

Accurate anatomical fracture reduction of tibial plateau fractures is often challenging since these fractures usually consist of multiple fragments which are displaced in different directions [1,2]. Surgical treatment consists of screw or plate osteosynthesis and aims to restore articular surface, achieve normal limb alignment, and re-establish joint stability [3,4]. Achieving these goals is believed to reduce the risk of post-traumatic osteoarthritis [5,6]. Most studies reporting on functional recovery after surgical treatment of tibial plateau fractures focus on achieved surgical reduction and their relationship with functional outcome. Yet initial displacement is often neglected. The exact impact of both initial fracture displacement and the quality of operative fracture reduction on patient-reported outcome remains a matter of debate [7].

Both the severity of the fracture as well as the postoperative quality of reduction are assessed by evaluating fracture displacement. Displacement is measured in terms of the maximal gap and step-off on a single coronal, sagittal, or axial CT slice. This method is, however, known for its high inter- and intraobserver variability [8,9]. Moreover, it tends to underestimate fracture displacement and does not provide a full representation of the articular incongruity [8,10]. Assessment of fracture displacement unfortunately relies on which CT slice is selected for measurement and by whom it is measured. This complicates any study which addresses the association between fracture displacement and the functional outcome at follow-up [9].

Recently, we introduced a Quantitative 3D CT (Q3DCT) method to quantify the fracture displacement in tibial plateau fractures. This method introduces the 3D gap area, which represents a full quantification of the intra-articular incongruity and shows less user dependency compared to conventional 2D measurements of fracture displacement [8]. In addition, a recent study showed that this measurement is predictive for risk for conversion to a total knee arthroplasty at follow-up [11]. The aim of this study is to assess the association between fracture displacement and mid-term functional recovery. Our research questions were: (1) What is the association between the initial fracture displacement, as measured in 3D on the preoperative CT scan, and the patient-reported functional outcome at follow-up? (2) What is the association between the residual fracture displacement, as measured in 3D on the postoperative CT scan, and the patient-reported functional outcome at follow-up? Our hypothesis is that both initial and residual fracture displacement is associated with patient-reported functional outcome.

## 2. Materials and Methods

### 2.1. Study Design

A multicentre cross-sectional study was performed within three hospitals (one level 1 and two level 2 trauma centres). All patients who had been treated surgically for a tibial plateau fracture between 2003 and 2018 were identified. Patients were eligible for inclusion based upon the availability of a preoperative CT scan of the injured knee with a slice thickness of ≤1 mm. Patients’ demographics were retrieved from their electronic records, and we verified whether they were still alive using the population registry. Fracture classification was performed according to the AO/OTA system [12]. Patients with an isolated tibial eminence avulsion, a complicated fracture requiring amputation of the injured leg, age <18 years, and those deceased or with an unknown address at the time of follow-up were excluded.

### 2.2. Participants

A total of 766 patients were treated surgically for a tibial plateau fracture, of which 4 had an amputation, 33 were <18 years, 74 had died at follow-up, and 12 had an unknown address. An additional 6 patients had to be excluded due to insufficient quality of the postoperative images, leaving 637 eligible patients for follow-up analysis. All patients were approached by posted mail, of which 362 responded at a mean follow-up of 7.0 ± 3.7 years (57% response rate). Patient demographics are described in Table 1.

### 2.3. Patient-Reported Outcome

All eligible patients were approached by posted mail and asked to provide informed consent and complete the validated and standardized Knee Injury and Osteoarthritis Outcome Scale (KOOS) questionnaire using the Dutch language [13]. KOOS is a questionnaire designed to assess short- and long-term patient-relevant outcomes following knee injuries. It contains 42 items in 5 separately scored subscales: pain, symptoms, activities of daily living (ADL), function in sport and recreation (sport), and quality of life (QoL). Scores for the subscales were calculated by adding the individual items (questions) and transforming scores to a range from 0 to 100, with higher scores indicating better function. Patients who underwent conversion to total knee arthroplasty (TKA) were assigned an average KOOS score as it would have been just before conversion to TKA was performed. The assigned score was retrieved from a previous cohort [14]. The assumed KOOS subscores were 52 for symptoms, 45 for pain, 55 for ADL, 16 for sport, and 27 for QoL. The rationale for doing this was that the KOOS should represent the situation as it was just before conversion to TKA, since this corresponds to the complaints that arose from the initial and residual displacement.

### 2.4. Three-Dimensional Assessment of Initial (Preoperative) and Residual (Postoperative) Fracture Displacement

#### 2.4.1. Initial Fracture Displacement

**Figure 1:**
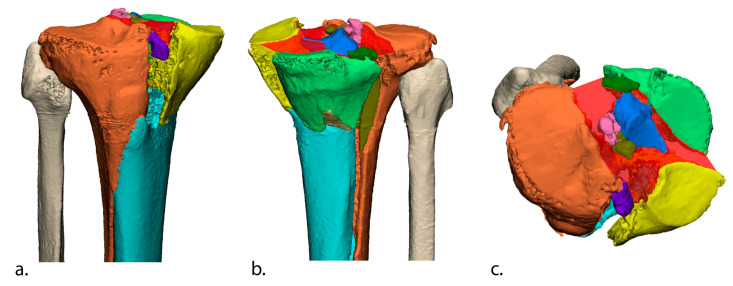
The 3D gap area (red surface) measurement on a 3D fracture model representing total fracture displacement between all fracture fragments at the articular level. All different fracture fragments were assigned a different colour. The 3D gap area is depicted from the anterior (**a**), posterior (**b**), and cranial (**c**) view.

The data from the preoperative CT scan of each patient were used to create a 3D fracture model. CT data (DICOM files, Digital Imaging and Communications in Medicine) were imported into Mimics software (Version 23.0, Materialise, Leuven, Belgium), in which a segmentation process was performed, in which all fragments were segmented. A 3D assessment of the initial fracture displacement was performed for each patient by measuring the 3D gap area according to our previously published method: (1) delineating the articular surface; (2) extracting the fracture lines from the contours of the articular surface; and (3) measuring a 3D surface between all these fracture lines [11]. The 3D gap area represents the distances between all fracture lines in all planes, and the created surface (mm^2^) is considered a quantitative measure of the initial fracture displacement between all fracture fragments (Figure 1).

#### 2.4.2. Residual Fracture Displacement

**Figure 2:**
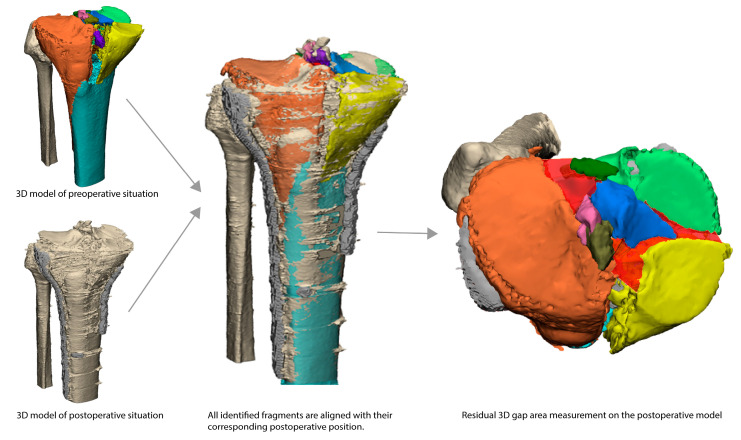
Residual 3D gap area (red area) is measured after all identified fragments (all assigned a different colour) are positioned according to the postoperative situation.

A postoperative CT scan was only available in some cases. It was not part of the standard of care, and the decision to perform a CT scan was based on the clinical judgement of the treating surgeon. The main reason for performing a postoperative CT scan was dissatisfaction with the fracture reduction on the postoperative radiograph. In order to assess the residual fracture displacement, the postoperative CT data (DICOM) were imported into the Mimics software (Version 23.0, Materialise, Leuven, Belgium), in which a segmentation process was performed. A preset bone threshold range (Hounsfield Unit 226-2500) was used, combined with the ‘region growing’ function, to separate the tibia from the implant(s), screws, and femur. The segmentation was checked and manually brushed to correct for the artefacts resulting from the implants and screws. After segmentation, both the pre- and postoperative 3D models were imported into 3-matic medical software (Version 15.0, Materialise, Leuven, Belgium). To subsequently measure the residual 3D gap area, the preoperative fracture fragments were matched with the postoperative 3D model using surface-based matching to avoid the possible influence of metal artefacts (Figure 2). The 3D gap area was measured on the (preoperative) fragments positioned on their positions after surgery. For this measurement, the same method as applied preoperatively was used.

### 2.5. Postoperative Evaluation

Poor articular reduction and tibial alignment are associated with worse functional outcome [15,16,17]. In order to correct for the quality of the reduction in patients without a postoperative CT scan, the quality of the fracture reduction and the tibial alignment were evaluated on the postoperative radiographs using three radiographic parameters: articular fracture reduction, coronal alignment, and sagittal alignment. Articular fracture reduction was assessed by measuring the maximum residual intra-articular incongruence (gap and step-off). Coronal alignment was assessed by measuring the medial proximal tibial angle (MPTA) on the anteroposterior radiograph, whereas sagittal alignment was assessed by measuring the posterior proximal tibial angle (PPTA) on the lateral radiograph. The fracture reduction was considered as “anatomical” when both the gap and step-off were ≤ 2 mm, when the MPTA was 87 ± 5°, and the PPTA was 9 ± 5 [18,19].

### 2.6. Primary and Secondary Study Goals

The primary study goal was to assess the association between initial fracture displacement and the patient-reported outcome at follow-up. To achieve this, we measured the initial displacement in terms of 3D gap area on the 3D reconstruction of the initial CT scan and related this to a validated patient-reported outcome at follow-up. To correct for the quality of reduction, the articular reduction and the tibial alignment were evaluated on the postoperative radiographs using three radiographic parameters: articular fracture reduction, coronal alignment, and sagittal alignment.

The secondary study goal was to assess the association between residual fracture displacement and the patient-reported outcome at follow-up. To achieve this, we performed a subanalysis in patients with available postoperative CT scans. The residual displacement in terms of 3D gap area was measured in these patients on the 3D reconstruction of the postoperative CT scan and was related to validated patient-reported outcome at follow-up.

### 2.7. Statistical Analysis

Statistical analysis was performed using SPSS (version 28, IBM, Chicago, IL, USA). Continuous variables are presented as the mean with standard deviation for normally distributed data and median with interquartile range for non-normally distributed data. Descriptive statistics are used to describe the study population. The study population was divided into groups based on the size of the initial and residual 3D gap area. These prognostic groups were identified in our previous research and were excellent (gap area: 0–150 mm^2^), good (151–550 mm^2^), moderate (551–1000 mm^2^), and poor (>1000 mm^2^) [11]. Analysis of variance (ANOVA) was used to assess differences between these groups in terms of functional outcome. Multiple linear regression was performed to assess the association between initial and residual fracture displacement and the patient-reported outcome. The five subscales of the KOOS questionnaire were the outcomes of interest (dependent variable). Two potential predictors (initial and residual 3D gap area) were assessed within two separate regression models. A total of eight potential confounders (age, gender, BMI, smoking, AO/OTA classification, complication, inadequate reduction on postoperative radiograph, and follow-up time) were included in both models. A *p*-value of less than 0.05 was considered statistically significant.

### 2.8. Analysis of Nonresponders

Nonresponse analysis demonstrated no differences in age (52.7 ± 14.0 vs. 48.9 ± 16.9; *p* = 0.207) between responders and nonresponders. Responders were more often women compared to nonresponders (69.6% vs. 59.7%; *p* = 0.031).

## 3. Results

### 3.1. Association between Initial 3D Displacement and Functional Outcome

**Figure 3:**
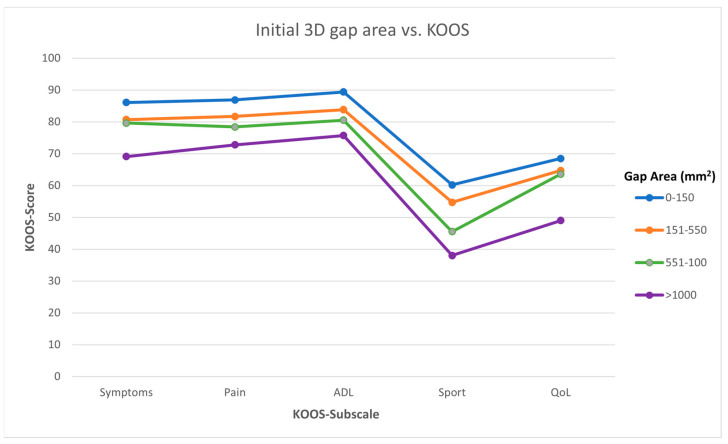
Assessment of the association between initial (preoperative) fracture displacement and functional outcome. The average scores of the KOOS subscales for the four groups, which were divided based on an increasing preoperative 3D gap area.

In a total of 55 patients, the initial gap area was <150 mm^2^; a total of 148 patients had a gap area between 151 and 550 mm^2^; a total of 72 patients had a gap area between 551 and 1000 mm^2^; and 87 patients had a gap of >1000 mm^2^. Functional outcome became worse when initial 3D gap area increased in all subscales of the KOOS questionnaire (Figure 3). In terms of symptoms, the KOOS value dropped from 86.1 ± 18.2 in the 0–150 group to 68.9 ± 24.6 in the >1000 mm^2^ group (*p* < 0.001). Similar results were seen in the pain (86.9 ± 16.8 to 72.6 ± 23.3, *p* = 0.002), ADL (89.4 ± 14.7 to 75.5 ± 23.0, *p* = 0.002), sport (59.8 ± 33.2 to 38.2 ± 32.1, *p* < 0.001), and QoL (68.5 ± 24.5 to 48.9 ± 26.3, *p* < 0.001) subscales.

Multivariate linear regression shows that the initial 3D gap area is a negative predictor for all subscales of the KOOS with correlation coefficients varying from −0.8 to −1.4 after correction for potential confounders (Table 2).

### 3.2. Association between Residual 3D Displacement and Functional Outcome

**Figure 4:**
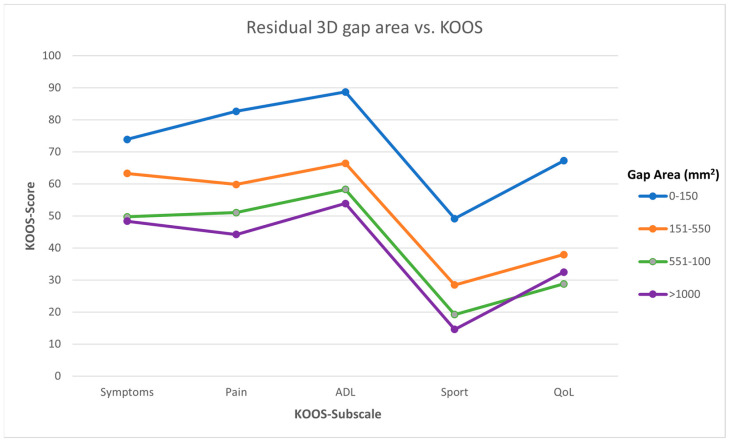
Assessment of the association between residual (postoperative) fracture displacement and functional outcome. The average scores of the KOOS subscales for the four groups, which were divided based on an increasing postoperative 3D gap area.

In 72 patients, a postoperative CT scan was available. A total of 11 patients had a gap area < 150 mm^2^, 31 between 151 and 550 mm^2^, 25 between 551 and 1000 mm^2^ and 5 > 1000 mm^2^. Patient-reported outcome as measured in all KOOS subscales became worse when the postoperative 3D gap area increased (Figure 4). In terms of symptoms, the KOOS value dropped from 73.9 ± 21.1 in the 0–150 group to 48.3 ± 16.4 in the >1000 mm^2^ group (*p* < 0.001). Similar results were seen in the pain (82.7 ± 18.2 to 44.3 ± 15.0, *p* = 0.001), ADL (88.7 ± 13.6 to 53.9 ± 17.2, *p* = 0.001), sport (49.2 ± 30.1 to 14.6 ± 8.1, *p* < 0.001), and QoL (67.2 ± 23.4 to 32.5 ± 11.9, *p* < 0.001) subscales. Multiple linear regression models showed that the residual 3D gap area had a negative association with the symptoms, ADL, sport, and quality of life subscale of KOOS with correlation coefficients varying from −2.2 to −2.6 (Table 2). The full regression models for both initial and residual 3D displacement can be found in the Appendix A and Appendix B.

## 4. Discussion

**Figure 5:**
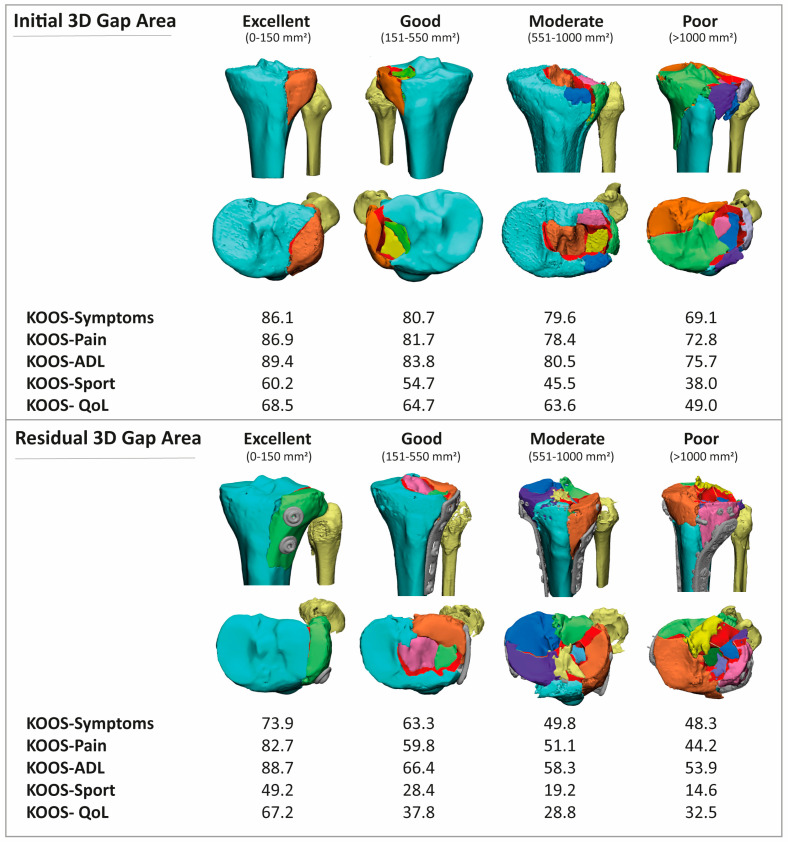
Overview of the different prognostic groups, represented by 3D fracture models (gap area indicated in red, all fragments assigned a different colour) and their associated average KOOS value.

This study shows that both increasing initial and residual displacement, as measured using a validated 3D measurement technique, are negatively associated with the patient-reported functional outcome. These results indicate that part of the functional outcome might already be determined by the irreversible damage to the articular surface caused by the initial trauma. Yet, patients benefit from the quality of the reduction, which is positively associated with a patient’s outcome. Assigning patients based on their 3D gap area into four different prognostic groups (excellent, good, moderate, and poor), shows that the higher the 3D gap area, the lower average KOOS values (Figure 5). This provides a tool which could potentially be used for patient counselling regarding their expected functional outcome.

Irreversible damage to the articular surface and the ligamentous structures of the knee joint caused by the initial trauma contributes to worse functional outcome despite adequate articular fracture reduction [9,20]. However, only a limited number of studies have reported on the relationship between severity of the trauma and functional recovery of the patient. Recently, Parkkinnen et al. showed that initial articular depression measured from preoperative CT scans was a significant predictor of the development of osteoarthritis [21]. In addition, our research group recently showed that initial displacement, as measured in 3D, is independently associated with the development of severe osteoarthritis with the need for TKA [11]. No studies, however, have reported on the actual relationship of the initial displacement and the patient-reported outcome. The current study is the first study that addresses this association and shows that the initial 3D fracture displacement is independently associated with functional recovery in terms of the subscales symptoms, all day activities, sport, and quality of life. Our results indicates that with every 100 mm^2^ increase in the preoperative gap area, the KOOS value decreases up to 1.4 points. With gap area sizes observed up to 3000 mm^2^, this can lead to a clinically significant change in outcome [22]. This shows that despite good anatomical reduction, the initial fracture displacement is already a predictor of outcome for the patient. This is of importance for a full understanding of these fractures and patient counselling.

This study shows that not only initial but also residual 3D fracture displacement affects the functional outcome. Our results indicate that with every 100 mm^2^ increase in the residual gap area, the KOOS value decreases up to 2.6 points. This shows that the residual displacement has a strong impact on the functional outcome and emphasizes the need for accurate reduction of the articular surface. Various authors have already demonstrated the importance of articular reduction on functional outcome by showing that a postoperative gap and step-off of more than 2 mm is associated with worse functional outcome or the development of osteoarthritis [5,19,21]. However, all these measurements were performed on plain postoperative radiographs, which are often an underestimation of the truth gap or step-off [23,24]. Singleton et al. recently assessed postoperative reduction on a CT scan and showed that a step-off of less than 2.5 mm was associated with a better functional outcome [15]. These observations, together with our findings, underscore the need for accurate articular reduction. Measuring the displacement in 3D solves the problems with observer reliability. In addition to previous research, this provides a reliable quantification of the impact of displacement and the association with functional recovery. For clinical use, patients could, for instance, be placed in the four proposed prognostic groups (excellent, good, moderate, and poor) with their corresponding expected functional outcome. With that, it could guide the physician in providing the patient with a personalized estimation of prognosis. Yet, performance in other clinical contexts should be performed to ensure validity.

This study has several limitations: (1) Selection bias is inherent to our cross-sectional cohort study, caused by loss to follow-up and nonresponse to the sent questionnaire. Nonresponse analysis indicated that responders were more often women. Yet, no significant differences in age were found between responders and nonresponders. (2) There was a high variation in follow-up duration (7.0 ± 3.7 months), which is inherent to a cross-sectional study design. To correct for this, we included follow-up time as a potential confounder in the analysis. (3) The number of patients with a postoperative CT scan was limited because it was not part of the standard of care. These patients had a postoperative CT due to a clinical suspicion of inadequate fracture reduction, which introduces selection bias for the assessment of residual displacement. However, despite potential bias, the results indicate a clear association between increasing postoperative fracture displacement and worse functional outcome. Given the limited number of available postop CT scans, our findings regarding the association between 3D residual fracture displacement and patient-reported outcome can only be considered hypothesis-generating and not prescriptive. (4) Another important practical limitation is that performing the 3D fracture assessments is labour-intensive. Depending on the fracture comminution, the segmentation and measurement process can take up to one hour. The 3D fracture assessment of the initial displacement should therefore be reserved for selected cases. Before widespread implementation in clinical practice, further automatization of the 3D measurements is recommended.

## 5. Conclusions

In this study, a novel 3D measurement method, which is not subject to problems with interobserver reliability, was applied to quantify the initial and residual displacement. This is the first study that could reliably classify the degree of displacement and shows that increasing displacement results is poorer patient-reported functional outcomes. Potentially, these measurements could guide the physician in providing the patient with personalized estimation of the prognosis. Large, prospective, cohort studies with the availability of pre- and postoperative CT scans are needed to assess the truth relationship between the degree of 3D fracture displacement and functional recovery after surgical treatment of tibial plateau fractures.

## Figures and Tables

**Table 1:** Patient characteristics (*n* = 362).

Parameter	Value
Age in years	52 (±14)
Women	250 (69%)
BMI in kg/m^2^	26.2 (±4.7)
Smoking	83 (23%)
AO/OTA classification	
41-B1	19 (5%)
41-B2	58 (16%)
41-B3	195 (54%)
41-C1	22 (6%)
41-C2	9 (3%)
41-C3	59 (16%)
Surgical treatment	
Plate osteosynthesis	294 (81%)
Screw osteosynthesis	68 (19%)
Complication	
Infection	21 (6%)
Malunion	11 (3%)
Meniscal or ligamental reconstruction	6 (2%)
Nerve damage	2 (1%)
Compartment syndrome	1 (0%)
Follow-up (years)	7.0 (±3.7)
Conversion to total knee arthroplasty	51 (14%)

**Table 2:** Multiple linear regression models for initial and residual 3D gap area associated with the different KOOS subscales.

	Initial 3D Gap Area (×100) (n = 362) *		Residual 3D Gap Area (×100) (n = 72) **	
	B (95% CI)	*p* Value	B (95% CI)	*p* Value
KOOS-Symptoms	−0.9 (−1.3 to −0.5)	<0.001	−2.2 (−3.9 to −0.5)	0.011
KOOS-Pain	−0.9 (−1.4 to −0.5)	<0.001	−2.4 (−4.4 to 0.2)	0.17
KOOS-ADL	−0.8 (−1.2 to −0.4)	0.002	−2.2 (−3.9 to −0.0)	0.014
KOOS-Sport	−1.4 (−2.1 to −0.7)	<0.001	−2.6 (−5.0 to −0.2)	0.033
KOOS-QoL	−1.1 (−1.7 to −0.5)	<0.001	−2.4 (−4.5 to −0.3)	0.023

* Included confounders: age, sex, BMI, smoking, AO/OTA classification, complication, inadequate reduction on postoperative radiograph, and follow-up time. ** Included confounders: age, sex, BMI, smoking, AO/OTA classification, complication, and follow-up time.

## Data Availability

The authors declare that the data supporting the findings of this study are available within the paper.

## Appendix A. Full Regression Model for Initial 3D Displacement in Relation to KOOS Functional Outcome

**Table:**

	KOOS-Symptoms (R^2^ = 0.22, adj R^2^ = 0.20)		KOOS-Pain (R^2^ = 0.21, adj R^2^ = 0.19)		KOOS-ADL (R^2^ = 0.22, adj R^2^ = 0.20)		KOOS-Sport (R^2^ = 0.18, adj R^2^ = 0.16)		KOOS-QoL (R^2^ = 0.18, adj R^2^ = 0.16)	
	B (95% CI)	*p* Value	B (95% CI)	*p* Value	B (95% CI)	*p* Value	B (95% CI)	*p* Value	B (95% CI)	*p* Value
Preoperative 3D gap area (×100)	−0.9 (−1.3 to −0.5)	<0.001	−0.9 (−1.4 to −0.5)	<0.001	−0.8 (−1.2 to −0.4)	0.002	−1.4 (−2.1 to −0.7)	<0.001	−1.1 (−1.7 to −0.5)	<0.001
Age	0.2 (0.0 to 0.4)	0.016	0.1 (−0.1 to 0.2)	0.452	−0.1 (−0.2 to 0.1)	0.429	0.1 (−0.2 to 0.3)	0.482	0.1 (−0.1 to 0.3)	0.221
Male	5.2 (0.7 to 9.7)	0.027	6.3 (1.5 to 11.1)	0.010	6.3 (2.0 to 10.7)	0.004	12.1 (4.9 to 19.3)	0.001	4.6 (−1.3 to 10.6)	0.127
BMI	−0.7 (−1.1 to −0.3)	0.002	−0.9 (−1.4 to −0.5)	<0.001	−1.0 (−1.4 to −0.5	<0.001	−1.4 (−2.1 to −0.7)	<0.001	−0.9 (−1.4 to −0.3)	0.004
Smoking	−4.0 (−8.8 to 0.8)	0.104	−6.5 (−11.7 to −1.3)	0.014	−7.1 (−11.8 to −2.4)	0.003	−6.3 (−14.1 to 1.5)	0.115	−5.6 (−12.0 to 0.9)	0.090
AO/OTA	−0.8 (−2.4 to 0.9)	0.374	−0.3 (−2.1 to 1.5)	0.714	−0.7 (−2.3 to 0.9)	0.410	−1.6 (−4.2 to 1.1)	0.256	−0.8 (−3.0 to 1.4)	0.494
Postoperative complication	−9.9 (−16.3 to −3.4)	0.003	−9.8 (−16.8 to −2.9)	0.006	−8.9 (−15.2 to −2.6)	0.006	−9.1 (−19.4 to 1.3)	0.086	−11.2 (−19.9 to −2.6)	0.011
Nonanatomical reduction	−5.7 (−10.3 to −1.0)	0.016	−5.0 (−10.0 to −0.5)	0.048	−2.8 (−7.3 to 1.7)	0.223	−4.6 (−12.2 to 2.9)	0.229	−7.4 (−13.6 to −1.2)	0.018
Follow-up time (years)	0.7 (0.1 to 1.2)	0.018	0.5 (−0.1 to 1.1)	0.089	0.5 (−0.1 to 1.0)	0.081	0.3 (−0.6 to 1.2)	0.548	0.8 (0.0 to 1.5)	0.045

## Appendix B. Full Regression Model for Residual 3D Displacement in Relation to KOOS Functional Outcome

**Table:**

	KOOS-Symptoms (R^2^ = 0.28, adj R^2^ = 0.19)		KOOS-Pain (R^2^ = 0.27, adj R^2^ = 0.17)		KOOS-ADL (R^2^ = 0.31, adj R^2^ = 0.21)		KOOS-Sport (R^2^ = 0.20, adj R^2^ = 0.09)		KOOS-QoL (R^2^ = 0.27, adj R^2^ = 0.17)	
	B (95% CI)	*p* Value	B (95% CI)	*p* Value	B (95% CI)	*p* Value	B (95% CI)	*p* Value	B (95% CI)	*p* Value
Postoperative 3D gap area (×100)	−2.2 (−3.9 to −0.5)	0.011	−2.4 (−4.4 to −0.5)	0.17	−2.2 (−3.9 to −0.0)	0.014	−2.6 (−5.0 to −0.2)	0.033	−2.4 (−4.5 to −0.3)	0.023
Age	−0.2 (−0.5 to 0.2)	0.360	−0.4 (−0.8 to −0.0)	0.046	−0.4 (−0.7 to −0.0)	0.044	−0.2 (−0.7 to 0.3)	0.418	−0.2 (−0.6 to 0.0)	0.361
Male sex	6.1 (−4.0 to 16.2)	0.232	2.6 (−9.3 to 14.6)	0.659	2.1 (−8.3 to 12.5)	0.687	2.5 (−11.7 to 16.8)	0.723	−4.8 (−17.3 to 7.8)	0.449
BMI	−0.4 (−1.2 to 0.5)	0.367	−0.3 (−1.3 to 0.6)	0.487	−0.4 (−1.2 to 0.5)	0.383	0.0 (−1.1 to 1.2)	0.938	0.3 (−0.7 to 1.4)	0.505
Smoking	−6.3 (−16.6 to 3.8)	0.216	−2.5 (−14.6 to 9.5)	0.675	−1.9 (−12.4 to 8.6)	0.723	−0.8 (−15.2 to 13.6)	0.912	−3.5 (−16.1 to 9.2)	0.588
AO/OTA	−1.1 (−4.3 to 2.0)	0.479	−1.2 (−4.9 to 2.6)	0.532	−1.3 (−4.6 to 1.9)	0.424	−2.1 (−6.6 to 2.2)	0.339	−2.9 (−6.9 to 1.0)	0.143
Postoperative complication	−4.7 (−14.8 to 5.7)	0.361	−2.3 (−14.3 to 9.6)	0.698	−3.4 (−13.8 to 7.0)	0.513	5.6 (−8.7 to 20.0)	0.433	−1.8 (−14.4 to 10.8)	0.773
Follow-up time (years)	0.3 (−1.0 to 1.6)	0.641	−0.6 (−2.1 to 0.9)	0.448	0.4 (−0.9 to 1.7)	0.557	0.2 (−1.6 to 2.0)	0.829	0.2 (−1.4 to 1.8)	0.798
